# Targeting ferroptosis as novel therapeutic approaches for epilepsy

**DOI:** 10.3389/fphar.2023.1185071

**Published:** 2023-04-13

**Authors:** Yuzi Jin, Lei Ren, Xiaoqing Jing, Hongquan Wang

**Affiliations:** ^1^ Department of Pediatrics, Affiliated Hospital of Chengde Medical University, Chengde, Hebei, China; ^2^ Department of Pancreatic Cancer, Tianjin Medical University Cancer Institute and Hospital, National Clinical Research Center for Cancer, Key Laboratory of Cancer Prevention and Therapy, Tianjin’s Clinical Research Center for Cancer, Tianjin, China

**Keywords:** epilepsy, seizure, ferroptosis, neuroprotection, treatment

## Abstract

Epilepsy is a chronic disorder of the central nervous system characterized by recurrent unprovoked seizures resulting from excessive synchronous discharge of neurons in the brain. As one of the most common complications of many neurological diseases, epilepsy is an expensive and complex global public health issue that is often accompanied by neurobehavioral comorbidities, such as abnormalities in cognition, psychiatric status, and social-adaptive behaviors. Recurrent or prolonged seizures can result in neuronal damage and cell death; however, the molecular mechanisms underlying the epilepsy-induced damage to neurons remain unclear. Ferroptosis, a novel type of regulated cell death characterized by iron-dependent lipid peroxidation, is involved in the pathophysiological progression of epilepsy. Emerging studies have demonstrated pharmacologically inhibiting ferroptosis can mitigate neuronal damage in epilepsy. In this review, we briefly describe the core molecular mechanisms of ferroptosis and the roles they play in contributing to epilepsy, highlight emerging compounds that can inhibit ferroptosis to treat epilepsy and associated neurobehavioral comorbidities, and outline their pharmacological beneficial effects. The current review suggests inhibiting ferroptosis as a therapeutic target for epilepsy and associated neurobehavioral comorbidities.

## 1 Introduction

Epilepsy is a chronic disorder of the central nervous system (CNS) affecting approximately 65 million individuals worldwide (Devinsky et al., 2018; Patel et al., 2019; Akyüz et al., 2023), with a global lifetime prevalence of 7.6 per 1,000 individuals (Van Loo et al., 2022). Epilepsy is characterized by aberrant neuronal activity and recurrent unprovoked seizures as a result of excessive synchronous neuronal discharge. Some forms of epilepsy are more common, while others are rare. Multiple environmental and genetic factors are thought to contribute to common forms of epilepsy, whereas high-impact rare genetic variants have been associated with rarer forms of epilepsy (Fiest et al., 2017). Epilepsy can be classified into two broad categories, idiopathic or genetic, according to the suspected underlying cause. Epilepsy can occur as a result of acute brain injuries, such as neurotrauma, stroke, brain tumors, or status epilepticus, genetic mutation, CNS infection, metabolic disorders, and autoimmune diseases (Vezzani et al., 2019).

Seizures are caused by an acute pathological insult or lesion that leads to a series of changes in molecular, cellular, and physiological properties, including inflammation (Vezzani et al., 2011), blood-brain barrier (BBB) injury (van Vliet et al., 2007), neurodegeneration (Dingledine et al., 2014), and functional changes in ion channels, transporters, receptors, and enzymes involved in neurotransmission (Devinsky et al., 2013). Epilepsy ultimately leads to epileptogenesis, resulting in the damage or loss of neurons, synaptic reorganization, axonal sprouting, and structural and functional changes in glial cells, particularly microglia and astrocytes.

The mechanisms underlying neuronal loss during epilepsy include oxidative stress, inflammation, iron overload, and excitotoxicity (Fabisiak and Patel, 2022; Parsons et al., 2022; Ramos-Riera et al., 2023). Due to inadequately equipped with antioxidant defense systems, the mammalian brain is prone to oxidative damage, which can be attributed to the enrichment of neuronal membrane lipids with poly-unsaturated fatty acyl (PUFA) side-chains, especially docosahexaenoic acid (DHA, C22:6) (Halliwell, 2006), high oxygen consumption (approximately 20% of total oxygen supply), abundance of iron (Burdo and Connor, 2003; Zecca et al., 2004; Ward et al., 2014), generation of mitochondrial reactive oxygen species, and accumulation of peroxisomal hydrogen peroxide (H_2_O_2_) (Halliwell, 2006). Previous studies have also identified multiple paradigms in regulated cell death (RCD) based on neuronal loss after epilepsy, including pyroptosis, autophagy, necroptosis, and apoptosis (Galluzzi et al., 2018; Mao et al., 2019a). However, epileptogenesis and the molecular mechanisms underlying neuronal loss after epilepsy are not completely understood. Therefore, further investigation is required to gain insight into the pathogenesis of epilepsy and epileptogenesis and identify clinically meaningful and effective targets for the development of new therapies for epilepsy.

In recent years, ferroptosis, a non-apoptotic iron-dependent lipid peroxide (LPO)-driven form of RCD, has been gaining attention in epilepsy research (Cai and Yang, 2021; Akyüz et al., 2023). Since the first study to report that ferroptosis was involved in seizure-induced cell death in rats and the associated cognitive comorbidities of kainic acid (KA)-induced temporal lobe epilepsy (Ye et al., 2019), emerging evidence has confirmed a role for ferroptosis in the pathogenesis of epilepsy (Cai and Yang, 2021), with several compounds exerting therapeutic effects through the pharmacological inhibition of ferroptosis in experimental epilepsy models. In this review, we briefly describe the core molecular mechanisms underlying ferroptosis and their role in epilepsy. Furthermore, we outline the beneficial pharmacological effects of compounds that inhibit ferroptosis and highlight their potential use in the treatment of epilepsy and associated neurobehavioral comorbidities.

## 2 Molecular mechanisms of ferroptosis

**FIGURE 1:**
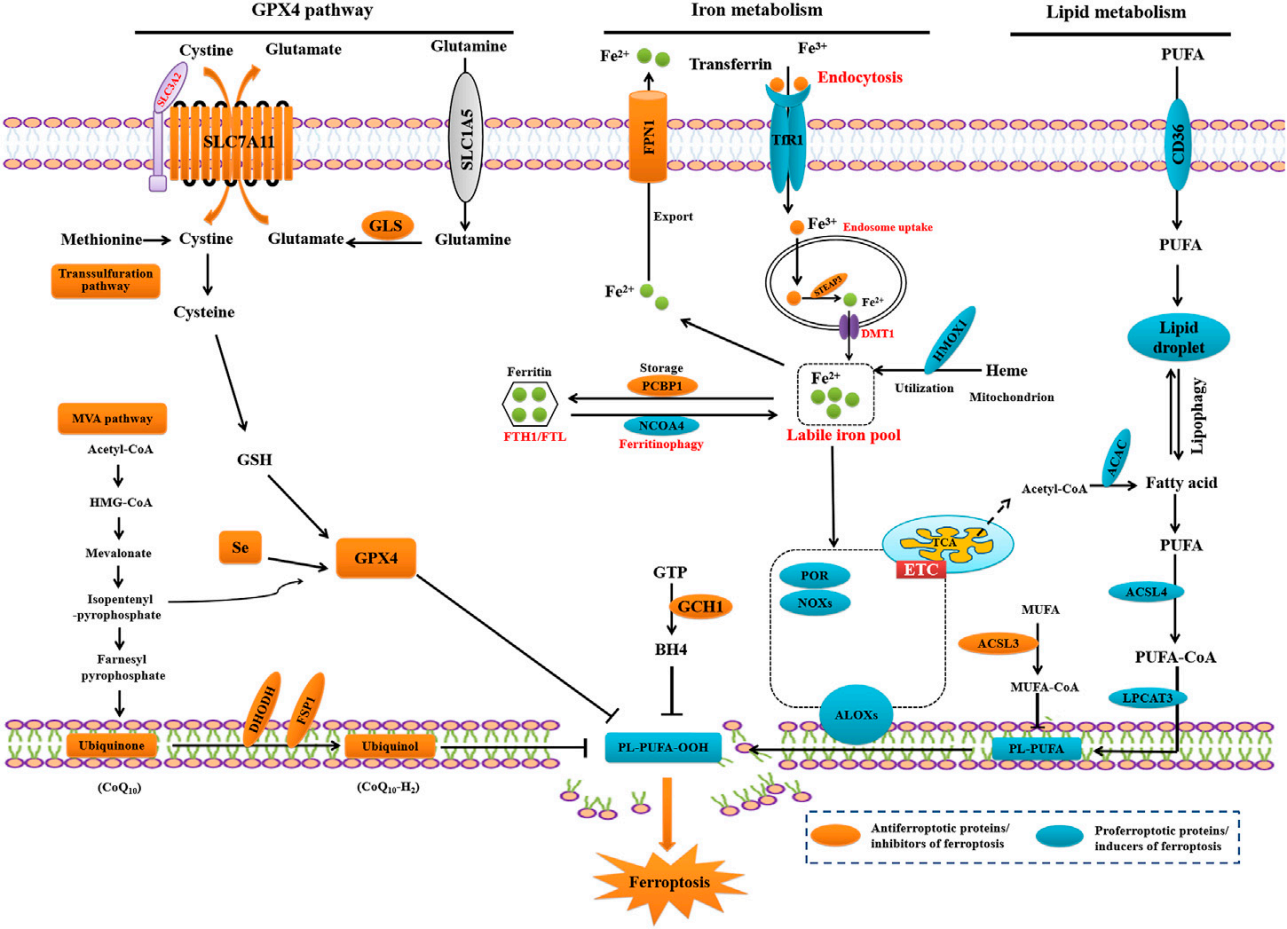
Core mechanisms of ferroptosis.

Ferroptosis occurs when ferroptosis-inducing factors, including iron-dependent reactive oxygen species (ROS) and LPOs, significantly override the antioxidant defense system, resulting in the lethal accumulation of LPO in cellular membranes, and ultimately membrane rupture (Tang and Kroemer, 2020; Lei et al., 2022). Two key signals, i.e., accumulation of free iron and inhibition of SLC7A11/GSH/GPX4 system are required to induce ferroptosis, as shown in Figure 1 (Chen et al., 2021).

### 2.1 SLC7A11-GSH-GPX4 axis

Cellular antioxidant systems that directly neutralize lipid peroxides comprise four anti-ferroptosis defense systems with distinct subcellular localizations. Ferroptosis defense systems include the SLC7A11/GSH/GPX4 (solute carrier family 7 member 11/glutathione/glutathione peroxidase 4), DHODH-CoQH_2_ (dihydroorotate dehydrogenase-dihydroubiquione), GCH1-BH4 (GTP cyclohydrolase 1-tetrahydrobiopterin), and FSP1-CoQ10 (ferroptosis suppressor protein 1-coenzyme Q10) pathways (Lei et al., 2022). SLC7A11/GSH/GPX4 is involved in amino acid metabolism and is a major cellular anti-ferroptosis defense system (Lei et al., 2022; Sun et al., 2022).

SLC7A11 and solute carrier family 3 member 2 (SLC3A2) make up the cystine/glutamate antiporter system Xc^−^. xCT, also known as xCT, is a core component of system Xc^−^ working as the transporter subunit of system Xc^−^, importing extracellular cystine and exporting intracellular glutamate to mediate the antiporter activity and (Sato et al., 1999; Koppula et al., 2018). The xCT-mediated uptake of extracellular cystine is promptly reduced to cysteine by a reduction reaction that consums nicotinamide adenine dinucleotide phosphate (NADPH) in the cytosol. Cysteine functions as a rate-limiting precursor for GSH biosynthesis, which is the principal cofactor for GPX4-mediated LPO detoxification (Koppula et al., 2021). As part of the GPX protein family, whose main biological role is protect lipids from peroxidation (Brigelius-Flohé and Maiorino, 2013; Brigelius-Flohé and Flohé, 2020), GPX4 has been identified as a key inhibitor of ferroptosis (Dixon et al., 2012; Friedmann Angeli et al., 2014; Yang et al., 2014; Ingold et al., 2018; Forcina and Dixon, 2019) and is regulated by epigenetic factors, transcription factors, and post-translational modifications (PTMs) (Cui et al., 2022). GPX4 simultaneously converts phospholipid hydroperoxides (PL-OOH) to non-toxic phospholipid alcohols and oxidizes two GSH molecules to oxidized glutathione (GSSG) (Ursini et al., 1982; Seibt et al., 2019). Previous studies have shown that the inhibition of SLC7A11 activity, or cystine depletion, in culture media can induce ferroptosis (Koppula et al., 2021).

### 2.2 Iron homeostasis

As an essential redox-active metal for neuronal activity and normal physiological function, iron plays a vital role in energy metabolism, biosynthesis, neurotransmission, and myelination (Crichton et al., 2011). Iron metabolism in humans is regulated through iron transport, absorption, and recycling. Iron homeostasis is highly modulated by iron regulatory proteins (IRPs), which include IRP1 and IRP2 (Piccinelli and Samuelsson, 2007; Muckenthaler et al., 2008; Ward et al., 2014), which bind to the iron response element (IRE) of iron-metabolizing genes and regulate their expression, leading to changes in cellular iron metabolism (Wang and Pantopoulos, 2011; Gao et al., 2019) and preventing the excessive accumulation of iron (Reichert et al., 2020; Sun et al., 2022). When intracellular iron is low, iron-sulfur (Fe-S) is released from the active site of the IRP, permitting IRP to bind to the IRE of divalentmetal-iontransporter-1 (DMT1) and transferrin receptor (TfR) gene transcripts to activate TfR translation, while IRPs bind to ferroportin (FPN) gene transcripts to inhibit FPN translation, resulting in the promotion of free iron absorption to increase the intracellular concentration and reduce the excretion of cellular iron (Reichert et al., 2020; Wei et al., 2020; Sun et al., 2022).

Iron (Fe^3+^) binds to circulating Tf to form the holotransferrin (holo-Tf) complex, (Ganz, 2008), which binds to transferrin receptor 1 (TfR1) and thereby enters brain microvascular endothelial cells (BMECs) through clathrin-mediated endocytosis (McCarthy and Kosman, 2012). TfR1, expressed on the luminal side of the BMECs, regulates the iron homeostasis in order to maintain proper brain function (Pardridge et al., 1987; Ward et al., 2014). The Fe^3+^-Tf-TfR complex is then transported into the endosome, where Fe^3+^ detaches from Tf and is reduced to Fe^2+^ by metalloreductases, duodenal cytochrome b (DCYTB) (Tulpule et al., 2010) or six-transmembrane epithelial antigen of prostrate 3 (STEAP3) (Ohgami et al., 2005). Fe^2+^ enters the cytosol via DMT1 (De Domenico et al., 2008). The unbound redox-active iron (Fe^2+^) in the cytosol forms a labile iron pool (LIP), which acts as an intermediate between stored, utilized, or imported iron, and feeds iron-catalyzed ROS production (Hassannia et al., 2021). Excess unbound Fe^2+^ can be exported to cells via FPN with the help of ceruloplasmin or hephaestin to oxidize Fe^2+^ and form Fe^3+^ (Andrews and Schmidt, 2007; Andrews, 2010). Alternatively, ferritin that consists of two isoforms: ferritin heavy (FTH) and ferritin light (FTL) stores unbound Fe^2+^ as non-reactive Fe^3+^. FTH exhibits activity of ferroxidase and is involved in rapid iron uptake and reutilization, while FTL take part in the long-term storage of iron through nucleation (Mills et al., 2010; Crichton et al., 2011; Singh et al., 2014; Ward et al., 2014). Ferritin can be degraded via ferritinophagy, an autophagy-like process that releases labile iron to facilitate LPO-mediated ferroptosis (Hadian and Stockwell, 2020). The utilized unbound Fe^2+^ can be transported to the mitochondria for heme biosynthesis, iron-sulfur cluster formation, and as a cofactor for mitochondrial enzymes.

Dysregulation and accumulation of free iron causes oxidative stress-dependent damage to neurons in the CNS, resulting in neurological diseases, including epilepsy (Chen S. et al., 2020). Free iron accumulation is involved in free radical formation and propagation of LPO, leading to the accumulation of lethal LPOs and the initiation of ferroptosis (Chen et al., 2021). Iron catalyzes the Fenton reaction, which induces the generation of free radicals that attack membrane-bound PUFAs in the non-enzymatic LPO pathway, while arachidonate lipoxygenases (ALOXs), the nonheme iron-containing enzymes, initiate the dioxygenation of membrane phospholipids containing PUFAs from linoleic acid (LA, 18:2 n-6) to arachidonic acid (AA, 20:4 n-6) in the enzymatic LPO pathway (Ryter et al., 2007; David et al., 2022). Alternatively, iron functions as an essential cofactor for cytochrome P450 oxidoreductase (POR) or ALOXs, both which promote the induction of LPO (Lei et al., 2022). The generated free radicals near the membrane react with PUFAs to produce lipid hydroperoxides, which are highly susceptible to degradation to form a variety of lipid derived α,β-unsaturated 4-hydroxyaldehydes, of which 4-hydroxynonenal (4-HNE) is the most prominent (Sayre et al., 2006; Schneider et al., 2008). Meanwhile, iron can promote the activity of iron-dependent peroxidases, including LOXs; thus, making cells are increasingly sensitive to ferroptosis (Chen X. et al., 2020).

Emerging studies have shown the levels of cell GSH and the labile iron pool are intimately linked (Chen J. et al., 2022). Reduced cysteine levels lead to the depletion of GSH levels and reduction of GPX4 levels and activity, resulting in the accumulation of unrepaired lipid peroxides and ferrous ions (Friedmann Angeli et al., 2014; Stockwell et al., 2017). GSH is a very important component of Fe^2+^ in unstable iron pools, it binds to Fe^2+^ to prevent lipid peroxidation, directly inhibiting GSH biosynthesis and triggering ferroptosis (Hider and Kong, 2011). GSH play a role in providing a ligand for the cytosolic iron pool (Hider et al., 2021). GSH depletion is one of the earliest detectable events in the substantia nigra (SN) of Parkinson’s disease, and a reduced levels of GSH in immortalized midbrain-derived dopaminergic neurons results in increases in the cellular labile iron pool (LIP). This increase is independent of either iron regulatory protein/iron regulatory element (IRP/IRE) or hypoxia inducible factor (HIF) induction but is both H_2_O_2_ and protein synthesis-dependent. This study suggest a novel mechanistic link between dopaminergic GSH depletion and increased iron levels based on translational activation of TfR1 (Kaur et al., 2009). Taken together, although the two pathways,i.e.,.SLC7A11-GSH-GPX4 axis and iron homeostasis, implicated in ferroptosis are indicated as separate, combined contribution of the two pathways in ferroptosis might vary in different systems.

### 2.3 Lipid peroxidation

Phospholipids with polyunsaturated acyl tails (PL-PUFAs) are the substrates for LPO during ferroptosis (Hadian and Stockwell, 2020) and are generated by acyl-coenzyme A (CoA) synthetase, long-chain family member 4 (ACSL4) and lysophosphatidylcholine acyltransferase (LPCATs). In the non-enzymatic LPO pathway, PUFAs are ligated with CoA by ACSL4 to produce acyl-CoA, which can be re-esterified into phospholipids by LPCATs. Iron initiates the non-enzymatic Fenton reaction and can be incorporated into ROS producing enzymes, acting as an essential cofactor for ALOXs and POR, which promote LPO, generating lipid peroxides or peroxidated PL-PUFAs(PL-PUFA-OOH), and eventually triggering cell death is the last step of ferroptosis (Hadian and Stockwell, 2020; Zou et al., 2020).

## 3 The role of ferroptosis in epilepsy

### 3.1 The role of ferroptosis in epilepsy-associated neuronal death

Ferroptosis has gained substantial attention and sparked great interest in the epilepsy research community in recent years. Given that ferroptosis has also been implicated in epilepsy pathogenesis, the pharmacological inhibition of ferroptosis may provide new therapeutic opportunities to treat this debilitating disease. Ferroptosis was first observed in the kainic acid-induced temporal lobe epileptic rat model (Ye et al., 2019), and was later corroborated in other studies and models of epilepsy (Mao et al., 2019b; Li et al., 2019). Mao et al. (2019a) demonstrated the induction of ferroptosis in mice models of pilocarpine (Pilo)and pentylenetetrazole (PTZ) kindling-induced seizures (Mao et al., 2019b). Previous indications that ferroptosis is involved in epilepsy were substantiated by hematological findings in children with epilepsy, which showed a consistent increase in 4-hydroxynonenal (4-HNE) and NAPDH oxidase 2 (NOX2), depletion of GSH, and inactivation of GPX4 (Petrillo et al., 2021).

Emerging evidence indicates that specific regulators modulate ferroptosis in epilepsy. For example, lysyl oxidase (LysOX) has been implicated in the pathogenesis of seizure-induced neuronal damage through extracellular signal-regulated protein kinase (ERK)-dependent 5-lipoxygenase (ALOX5) (Mao et al., 2022). In this study, lysOX promoted LPO in neurons by activating ERK-dependent ALOX5, which subsequently promotes ferroptosis. The enforced expression of LysOX increased the sensitivity of neurons to ferroptosis and aggravated seizure-induced hippocampal damage. Furthermore, inhibition of LysOX by β-aminopropionitrile (BAPN) significantly alleviated neuronal damage by preventing seizure-induced ferroptosis, revealing the LysOX-ERK-ALOX5 pathway as a target for ferroptosis regulation and therapeutic intervention (Mao et al., 2022). Another study has shown that circadian locomotor output cycles kaput (CLOCK) functions as an inhibitor of ferroptosis by increasing expression of GPX4 and peroxisome proliferator-activated receptor gamma (PPAR-γ) to protect against KA-induced seizures in mice (Wang et al., 2022). Knockout of CLOCK in mice exacerbated KA-induced seizures and reduced hippocampal expression of GPX4 and PPAR-γ in mice and N2a cells, while CLOCK overexpression inhibited ferroptosis by upregulating GPX4 and PPAR-γ. Together, this indicates that CLOCK inhibits GPX4 and PPAR-γ-dependent ferroptosis in KA-induced seizure mice.

### 3.2 The role of astrocyte activation-induced neuronal ferroptosis in epilepsy

Emerging evidence indicates that astrocytes, which are specialized neural cells of neuroepithelial origin, are crucial to the initiation and progression of epilepsy (Vezzani et al., 2022). Astrocytic activation is closely related to the emergence of ferroptosis in neurons. Neurotoxic A1 astrocytes mediate neuronal death by secreting neurotoxic long-chain saturated lipids (Guttenplan et al., 2021). A recent study revealed a correlation between astrocytic activation and neuronal ferroptosis in C57BL/6J male mice models of PTZ kindling-induced epileptic seizures and showed that A1 astrocytes induce ferroptosis via the chemokine receptor CXCL10/CXCR3 axis in epilepsy (Liang et al., 2023). Additionally, they found that ferroptosis inhibition prevented A1 astrocyte activation-induced neurotoxicity. CXCL10 increased the production of lipid ROS in a dose-dependent manner, which was blocked by the ferroptosis inhibitors ferrostatin-1 (Fer-1) and GSH in N2a cells. Similarly, NSC74859, a signal transducer and activator of transcription 3 (STAT3) inhibitor, reversed the CXCL10-induced downregulation of SLC7A11 and GPX4 and upregulation of lipid ROS. These findings suggest that A1 astrocyte-secreted CXCL10 activates STAT3 and suppresses SLC7A11/GPX4 in neurons via CXCR3, leading to ferroptosis (Liang et al., 2023). Clinical evidence also supported this correlation between A1 astrocytes and neuronal ferroptosis in patients with epilepsy. Therefore, A1 astrocyte activation-induced neuronal ferroptosis is a novel therapeutic target for epilepsy precision medicine.

### 3.3 The role of ferroptosis in epilepsy-associated cognitive deficits

A recent study showed that the anti-aging protein Klotho improved cognitive deficits in a rat model of temporal lobe epilepsy by inhibiting ferroptosis (Xiang et al., 2021). In a rat model of lithium chloride and pilocarpine (LiCl-Pilo)-induced temporal lobe epilepsy, Klotho overexpression effectively ameliorated cognitive deficits and prevented ferroptosis, as evidenced by the reduced iron accumulation, downregulated DMT1 expression, and increased FPN in the hippocampus. Furthermore, Klotho significantly increased GPX4 and GSH levels and suppressed ROS production (Xiang et al., 2021). In summary, Klotho exerted neuroprotection against epilepsy-associated cognitive deficits by inhibiting ferroptosis in a LiCl Pilo-induced temporal lobe epilepsy rat model.

## 4 Pharmacological inhibition of ferroptosis to treat epilepsy

**FIGURE 2:**
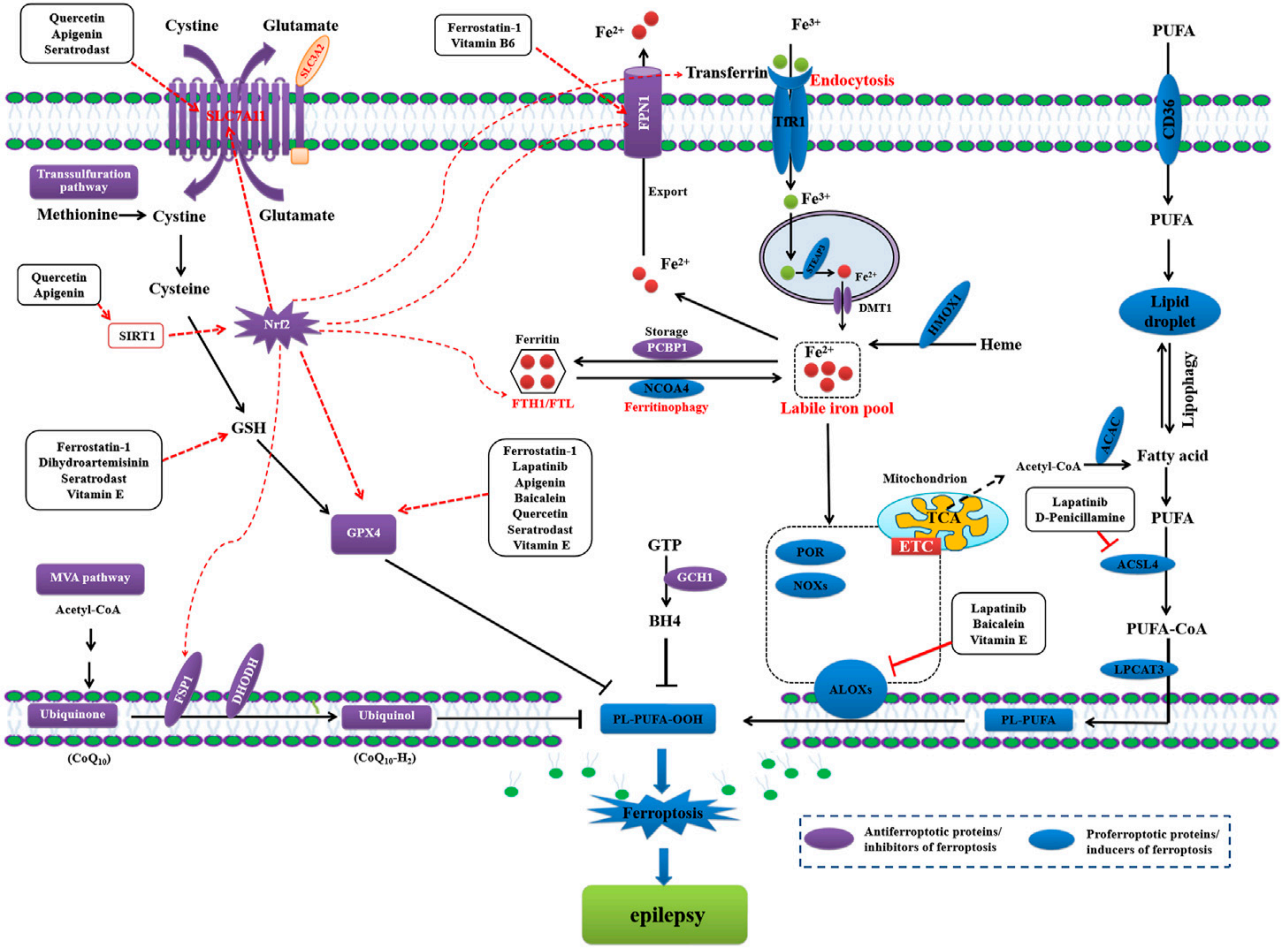
Pharmacological inhibition of ferroptosis to treat epilepsy.

Once ferroptosis was shown to play role in the pathogenesis of epilepsy, scientists immediately began exploring the possibility of treating epilepsy by pharmacologically inhibiting ferroptosis (Kahn-Kirby et al., 2019; Ye et al., 2019; Akyüz et al., 2023). Previously, inhibition of ferroptosis by Fer-1 prevented hippocampal neuronal loss and ameliorated cognitive impairment in KA-induced temporal lobe epilepsy in rats (Ye et al., 2019). Another report demonstrated the role of ferroptosis in the pathogenesis of mitochondrial disease-associated epilepsies and found that α-tocotrienol quinone (EPI-743) potently dose-dependently ameliorated ferroptosis in the cells of patient of five distinct pediatric epilepsy syndromes by reducing LPO and 15-hydroxyeicosatetraenoic acid (15-HETE), a specific 15-LO product (Kahn-Kirby et al., 2019). Baicalein has also been shown to significantly inhibit epilepsy in an iron chloride (FeCl_3_)-induced posttraumatic epilepsy mouse model and in ferric ammonium citrate (FAC)-induced HT22 hippocampal neuron cells by decreasing lipid ROS and 4-HNE, downregulating prostaglandin endoperoxide synthase 2 (PTGS2), and inhibiting the expression of 12/15-lipoxygenase (12/15-LOX). This suggests that baicalein exerts neuroprotection against posttraumatic epilepsy seizures by suppressing ferroptosis (Li et al., 2019). In both Pilo- and PTZ kindling-induced epileptic seizure mouse models, Fer-1 treatment potently ameliorated seizures by increasing the levels of GPX4 and GSH, inhibiting the production of 4-HNE and malondialdehyde, and decreasing accumulation of iron and the expression of *PTGS2* mRNA in the hippocampus (Mao et al., 2019a). Mounting evidence indicates that the pharmacological inhibition of ferroptosis exerts neuroprotective effects in *vitro* and *in vivo* epileptic disease models (Table 1; Figure 2).

**TABLE 1:** Emerging compounds targeted key regulators of ferroptosis to attenuate epilepsy.

Compounds	Experimental model	Epilepsy type	Findings	Mode of action	References
Quercetin	Kainic acid/Male C57BL/6J mice	Temporal lobe epilepsy	↓Seizure-like behaviors; ↓cognitive impairment; ↓ glutamate-induced HT22 neuronal cell death; ↑SIRT1/Nrf2/SLC7A11/GPX4	Nrf2/SLC7A11/GPX4	Xie et al. (2022)
GIF-0726-r	Glutamate/HT22 cells	-	↑Cell viability; ↓Lipd ROS; ↓ferrous ions	Ions	Hirata et al. (2021)
GIF-0726-r	Erastin/HT22 cells	-	↑Cell viability; ↓Lipd ROS; ↓ferrous ions	Ions	Hirata et al. (2021)
BAPN			↓Ferroptosis		Mao et al. (2022)
Ferrostatin-1	Kainic acid/rats	Temporal lobe epilepsy	↓Initiation and progression of ferroptosis in the hippocampus; ↑GPX4; ↑GSH; ↓lipid peroxides; ↓iron accumulation; ↓hippocampal neuronal loss; ↑cognitive function	GPX4	Ye et al. (2019)
Ferrostatin-1	Kainic acid/adult male SD rats	Temporal lobe epilepsy	↓Cognitive impairment; ↓activation of P38 MAPK; ↑synaptophysin; ↑postsynaptic density protein 95	P38 MAPK	Ye et al. (2020)
Lapatinib	Kainic acid/Male C57BL/6J mice	Temporal lobe epilepsy	↓Epileptic seizures; ↓seizure-induced hippocampal damage and electrical activity; ↓4-HNE; ↓PTGS2 (mRNA)	GPX4	Jia et al. (2020)
Lapatinib	Erastin/HT22 cells	Temporal lobe epilepsy	↑Cell viability; ↓lipid ROS; ↓4-HNE; ↓ MDA; ↑SLC7A11; ↑GPX4; ↓5-LOX; ↓ACSL4	GPX4	Jia et al. (2020)
Lapatinib	Glutamate/HT22 cells	Temporal lobe epilepsy	↑Cell viability; ↓ lipid ROS; ↓4-HNE; ↓ MDA; ↑SLC7A11; ↑GPX4; ↓5-LOX; ↓ACSL4	GPX4	Jia et al. (2020)
Dihydroartemisinin	Kainic acid/Balb/c nude mice	Temporal lobe epilepsy	↑TrxR; ↑GSH		Shao et al. (2022)
Apigenin	Kainic acid/Male C57BL/6J mice	Temporal lobe epilepsy	↓Epileptic symptoms; ↓oxidative stress; ↑SIRT1; ↓Ac-p53; ↑GPX4; ↑TrxR; ↑GSH	GPX4	Shao et al. (2020)
Apigenin	Kainic acid/SH-SY5Y	-	↓Intracellular superoxide; ↓lipid peroxidation accumulation; ↑GPX4	GPX4	Shao et al. (2020)
D-Penicillamine	Kainic acid/Male C57BL/6J mice	Temporal lobe epilepsy	↓Seizure-induced neuronal injury; ↑Neuronal survival; ↓ACSL4; ↓Ptgs2; ↓lipid peroxide	Aqp11/ACSL4	Yang et al. (2022)
D-Penicillamine	Glutamate or erastin/HT22 cells	-	↑Cell viability; ↓lipid ROS; ↓Ptgs2; ↓ACSL4; ↑Aqp11	Aqp11/ACSL4	Yang et al. (2022)
Baicalein	FeCl_3_/mouse	Posttraumatic epilepsy	↓Seizure score; ↓number of seizures; ↓average seizure duration; ↓4-HNE; ↓ 12/15-LOX	12/15-LOX	Li et al. (2019)
Baicalein	Ferric ammonium citrate/HT22 cells	-	↑Cell viability; ↓Lipd ROS; ↓4-HNE; ↑GPX4; ↓PTGS2	GPX4; PTGS2	Li et al. (2019)
Baicalein	Erastin/HT22 cells	-	↑Cell viability; ↓Lipd ROS; ↓4-HNE; ↑GPX4; ↓12/15-LOX	GPX4; 12/15-LOX	Li et al. (2019)
Ferrostatin-1	FeCl_3_/Male C57BL/6J mice	Posttraumatic epilepsy	↓Hippocampal damage; ↓seizure score; ↓spikes; ↓epileptiform discharge; ↓seizures per day; ↓the number of seizures; ↑seizure latency; ↓time in seizure; ↓epileptogenic progression; ↑cognitive deficits; ↓4-HNE; ↑GPX4	GPX4	Chen et al. (2022b)
Quercetin	Kainic acid/Male C57BL/6J mice	Temporal lobe epilepsy	↓Hippocampal damage; ↓seizure-like behaviors; ↓cognitive impairment; ↑SIRT1; ↑Nrf2; ↑SLC7A11; ↑GPX4; ↓morphological changes of mitochondria	Nrf2/SLC7A11/GPX4	Xie et al. (2022)
Quercetin	Glutamate/Erastin/HT22 cells	-	↑Cell viability; ↓4-HNE; ↓MDA; ↓lipid ROS; ↑SIRT1; ↑Nrf2; ↑SLC7A11; ↑GPX4	Nrf2/SLC7A11/GPX4	Xie et al. (2022)
Seratrodast	Pentylenetetrazole/Male C57BL/6J mice	Generalized clonic-tonic convulsions	↓Hippocampal damage; ↑ latency of the initial myoclonic jerks and GTCS; ↓seizure score; ↓seizure duration; ↑GPX4; ↓ p-JNK	GPX4	Hao et al. (2022)
Seratrodast	Erastin/HT22 cells	-	↑Cell viability; ↓ mitochondria damage; ↓lipid ROS; ↓ROS; ↓MDA; ↑SLC7A11; ↑GPX4; ↑GSH/GSSG; ↓p-JNK; ↓p53	SLC7A11/GPX4	Hao et al. (2022)
Vitamin E	Pentylenetetrazole/SD rat	Generalized clonic-tonic convulsions	↓Epileptic grade; ↓seizure latency; ↓number of seizures; ↓15-LOX; ↓MDA; ↓iron accumulation; ↑GPX4; ↑GSH	GPX4	Zhang et al. (2022)

12/15-LOX, 12/15-lipoxygenase; ACSL4, acyl-coA, synthetase long chain family member 4; ACSL4, long-chain acyl-CoA, synthetase 4; BAPN,β-aminopropionitrile; Nrf2, nuclear factor (erythroid-derived 2)-like 2; HO-1, haem-oxygenase-1; ACSL4, acyl-coA, synthetase long chain family member 4; ALOX5,5-lipoxygenase; MDA, malondialdehyde; GSH, glutathione; GSSG, oxidized glutathione; ALOX5, arachidonate 5-lipoxygena; Ptgs2,prostaglandin-endoperoxide synthase PTGS2, p synthase 2; TrxR, thioredoxin r.

## 5 Conclusions and perspectives

This review summarizes the recent progress in understanding of the pathological role and regulatory mechanisms of ferroptosis in epilepsy and highlights the use of ferroptosis inhibitors to reduce neuronal death and as a new therapeutic target for epilepsy. Ferroptosis is involved in epilepsy at three main stages: 1) ferroptosis-mediated epilepsy-associated neuronal death, 2) astrocyte activation-induced neuronal ferroptosis, and 3) ferroptosis-mediated epilepsy-associated cognitive deficits. However, given that current research investigating the role of ferroptosis in epilepsy is still in the initial stage, the specific mechanism by which ferroptosis orchestrates diverse cellular events in epilepsy is still poorly understood. First, the regulatory mechanism of ferroptosis in epilepsy needs to be elucidated. Second, identifying the diverse regulators of ferroptosis in epilepsy remains a challenge to be resolved. Third, although neuron-glia interactions have a clear role in the pathophysiology of epilepsy, whether other cells, such as oligodendrocytes, astrocytes, and microglia, are involved in modulating ferroptosis has yet to be determined. Lastly, recent investigations have been obtained from experimental studies that have a far-reaching clinical impact; thus, more clinical studies are needed. However, the potential drawbacks or limitations of the proposed pharmacological interventions such as ferrostatin as iron chelator causing systemic iron deprivation and thew role of multidrug resistance transporter P-glycoproteins that limit drug efficacy is uncertain (Lazarowski et al., 2007; Auzmendi et al., 2021). It remains an open conundrum for future investigate on whether inhibiting ferroptosis also renders drug resistance. Recent study have shown that P-glycoprotein confers resistance to ferroptosis inducers in cancer (Frye et al., 2023), suggesting that it is of fundamental importance to confirm whether P-glycoproteins play a role of confers drug resistance to ferroptosis inhibitors for epilepsy treatment. Nevertheless, pharmacological inhibition of ferroptosis is a promising therapeutic target for epilepsy.
